# Contamination of the Surfaces of a Health Care Environment by Multidrug-Resistant (MDR) Bacteria

**DOI:** 10.1155/2019/3236526

**Published:** 2019-11-29

**Authors:** Laila Chaoui, RajaaAit Mhand, Fouad Mellouki, Naima Rhallabi

**Affiliations:** ^1^Provincial Diagnostic Laboratory Epidemiological and Environmental Health, Provincial Health Delegation, Mohammedia, Morocco; ^2^Research Unit Microbiology Hygiene Bioactives Molecules Laboratory Virology Microbiology Quality and Biotechnology/Ecotoxicology Biodiversity, University Hassan II Casablanca, FSTM, Mohammedia, Morocco

## Abstract

Nosocomial infections (NIs) are known worldwide and remain a major problem despite scientific and technical advances in the field of health. The severity of the infection depends on the characteristics of the microorganisms involved and the high frequency of resistant pathogens in the hospital environment. The aim of this study is to determine the distribution of pathogenic bacteria (and their resistance to antibiotics) that spread on hospital surfaces, more specifically, on those of various departments in the Provincial Hospital Center (PHC) of Mohammedia, Morocco. A cross-sectional study was conducted from March 2017 to April 2018. Samples were collected by swabbing the hospital surfaces, and the isolated bacteria were checked for their susceptibility to antibiotics by the Kirby–Bauer disk diffusion method following the standards of the Clinical and Laboratory Standards Institute (CLSI). Among 200 swab samples, 176 (88%) showed bacterial growth. Gram-negative isolates were predominant at 51.5% (101/196), while the Gram-positives were at 48.5% (95/196). The main isolates are *Enterobacteria* weighted at 31.6% (62/196), *Staphylococcus aureus* reaching 24% (47/196), *Pseudomonas aeruginosa* at 9.2% (18/196), and *Acinetobacter* spp. with 3.3% (6/196). Moreover, the antimicrobial susceptibility profile of the isolates showed that about 31.7% (32/101) of the Gram-negative isolates were found to be MDR. This resistance is also high among isolates of *S*. *aureus* of which 44.7% (20/47) were methicillin-resistant *Staphylococcus aureus* (MRSA). Contamination of hospital surfaces by MDR bacteria is a real danger to public health. The concept of environmental bacterial reservoir is a reality that requires strict compliance with current guidelines and recommendations for hand hygiene, cleaning, and disinfection of surfaces in hospitals.

## 1. Background

Nosocomial infections (NIs) represent a major public health problem, with their frequency, severity, economic and social costs, and also the difficulty of controlling them. According to the World Health Organization (WHO), an average of 8.7% of hospital patients have NIs. At any given moment, it can be verified that over 1.4 million people worldwide suffer from infectious complications acquired in hospital [1]. Generally, NIs can be endogenous originate—the patient's own flora is the source of the infection, or exogenous—the pathogen comes from other patients, staff, or the hospital environment: water, air, or surfaces [2].

The contamination of surfaces depends on their characteristics, such as whether they are smooth, porous, or rough and/or on their state, such as whether they are dry, wet, new, or old. It also constitutes an ecological niche of bacteria capable of forming biofilms. These bacteria on many surfaces in a hospital last from a few days to long periods that can even go beyond 90 days [3]. Numerous investigations made in hospitals have highlighted the possible place of the inert environment as a contamination reservoir of patients, namely, because of the presence of multidrug-resistant bacteria (MDR) [4].

In Morocco, the incidence and prevalence of NIs are rarely published. A survey conducted in 1994 on an extended sample of 24 hospitals showed that the determined prevalence is 4.1% in provincial hospitals, 7.7% in regional hospitals, and 10.3% at the University Hospital Center (UHC) [5]. The prevalence of NIs at the Ibn Sina Hospital in Rabat is 9.5% and at the Ibn Rochd Casablanca Hospital Center it is 11.5%. Recently, this situation has become complicated by the emergence of multidrug-resistant bacteria, which poses a difficult task for clinicians who have limited therapeutic schemes [6].

These studies have focused on a number of bacterial species, such as methicillin-resistant *Staphylococcus aureus* (MRSA), glycopeptide-resistant *Enterococci* (GRE), *Acinetobacter baumannii*, and *Pseudomonas aeruginosa*. Bhalla et al. showed that the hands of caregivers who come into contact with only the colonized/infected patients' environment were often contaminated with bacteria, 30% of which were MRSA, 20% were GRE, and 15% were Gram-negative bacilli [7, 8]. This is a matter of a nonnegligible mode of contamination by manual transmission between environment, caregiver, and patient.

As part of the microbiological surveillance of the hospital environment, the present study represents a major focus on NI control strategies. Its aim is to determine, for the first time, the distribution of bacterial pathogens (resistant to antibiotics) that are prevalent on hospital surfaces of various departments in the Provincial Hospital Center (PHC) of Mohammedia. This enables the generation of local and original data on the emergence of MDR bacteria that could be the source of the spread of nosocomial diseases.

## 2. Materials and Methods

### 2.1. Framework and Research Timelines

A transverse bacteriological study was conducted from 1 March 2017 to 30 April 2018, in a public provincial hospital in Mohammedia city (Morocco), which had a 148-bed capacity and an average occupancy rate of 49.96 in 2016. The prefecture of Mohammedia covers an area of 277 km^2^. It is located between two of the most important cities in Morocco the economic city of Casablanca (25 km east) and the administrative city of Rabat (65 km west). The total population is 352,000 inhabitants (73% urban and 27% rural). All these characteristics make Mohammedia a city with specific health needs. These peculiarities guided the choice of the provincial hospital (the only one) of this city as the place for our study.

### 2.2. Study Site and Sampling

From five different departments (including the maternity, surgery, and medical wards, as well as the emergency and operating theatres), 241 surface samples were collected. Surface swab specimens were collected from predefined surfaces, such as operating tables, operating lights, beds, medical devices, floor, wall, and sinks, by using cotton swabs premoistened with sterile normal saline water according to ISO/DIS 14698-1 [9]. The choice of sampling points was made in consultation with the heads of departments and targeted the most critical and most representative locations in each department.

The collection was made in the early hours of the morning and transported in a cooler kept at 5 ± 3°C to be analyzed at the Provincial Epidemiological Diagnosis and Environmental Hygiene Laboratory (LPDEHM) under the authority of the provincial health department of Mohammedia.

### 2.3. Isolation and Phenotypic Identification of Aerobic Bacteria

After the delivery of samples to the laboratory, each swab was immersed in a liquid nutrient broth (BHI) and incubated at 37 ± 1°C for 24 h. Growth was noted, and the swabs were further subcultured in MacConkey agar, cetrimide agar, Slanetz agar, and salt agar. Then, they were incubated at 37 ± 1°C for 24 h. The characteristically distinct colonies were isolated and purified by subculturing in fresh media and incubating at 37 ± 1°C for 18–24 h to obtain pure culture isolates according to the recommendations of Meunier et al. [10].

The cultural, morphological, biochemical, and physiological characterizations of the bacterial isolates were determined by classical biochemical techniques and the API (bioMérieux, France) according to the schemes of Cheesbrough [11].

### 2.4. Antibiogram of Isolated Bacteria

The antimicrobial susceptibility tests of the isolates were determined by using the disk diffusion test according to the methods of Bauer et al. [12]. An inoculum of each isolate (approximately 1 × 108 cfu/ml) was developed by using the McFarland Standard and aseptically flooded on the surface of sterile Mueller–Hinton Agar (Oxoid, England).

Twenty antibiotic disks (Oxoid, England) were tested: penicillin (1 IU), gentamicin (10 *μ*g), kanamycin (30 *μ*g), erythromycin (15 *μ*g), ampicillin (10 *μ*g), amoxicillin-clavulanic acid (30 *μ*g), cefoxitin (30 *μ*g), ceftazidime (30 *μ*g), ceftriaxone (30 *μ*g), cefepime (30 *μ*g), tetracyclin (30 *μ*g), levofloxacin (5 *μ*g), imipenem (10 *μ*g), piperacillin (100 *μ*g), piperacillin/tazobactam (100/10 *μ*g), ticarcillin (75/10 *μ*g), sulfamethoxazol/trimethoprim (75/25 *μ*g), ciprofloxacin (5 *μ*g), chloramphenicol (30 *μ*g), and fusidic acid (10 *μ*g).

Antibiotics were selected on the basis of local availability, literature, effectiveness, and the guidelines of the Clinical and Laboratory Standards Institute (CLSI). They were aseptically placed on the seeded plates and then incubated at 37 ± 1°C for 18–24 h. The zone diameters of the drugs were measured and interpreted by using the criteria published by the CLSI [13]. Phenotypic detection of the presence of extended-spectrum beta-lactamase (ESBL) in Enterobacteria isolates was performed in vitro by the disk diffusion method also known as double-disk synergy test (a disk approximation test) combining Augmentin (amoxicillin-clavulanic acid) with a third-generation cephalosporin. The appearances of a synergistic image between these antibiotics (champagne cork) reflect a production of ESBL by the strain [13].

Resistance to methicillin among *S*. *aureus* strains was investigated using a cefoxitin disk under standard susceptibility testing. Strains with an inhibition diameter of less than 22 mm were considered MRSA [13].

## 3. Results

A total of 200 samples distributed as follows were analyzed: 50, floor and wall surfaces; 46, bed surfaces; 35, door and window handles; 24, equipment; 10, faucets and washbasins; 15, bedside tables; and 20 other sites. Of these samples, 176 (88%) showed positive cultures with 18 having mixed growth; 196 isolates were identified.

### 3.1. Distribution of Isolated Bacteria

From a bacteriological point of view, the positive threshold per department was 95% (38/40) in medicine, 92.5% (37/40) in maternity, 90% (36/40) in surgery, 87.5% (35/40) in emergencies, and 75% (30/40) in operating rooms. Various bacterial pathogens have been isolated as shown in Table 1.

A predominance of Gram-negative bacteria (GNB) with 101 isolates (51.5%) of which 61.4% (62/101) are *Enterobacteriaceae* is distributed as follows: 30.6% (19/62) of *Enterobacter* spp., 29% of *Klebsiella* spp. (18/62), 16.1% (10/62) of *Serratia* spp., 12.9% (8/62) of *Proteus* spp., 8.1% (5/62) of *Citrobacter* spp., and 3.2% (2/62) are *E*. *coli* species. The other GNB are represented by the species of *Acinetobacter* with an incidence of 5.9% (6/101) and 17.8% (18/101) of *Pseudomonas aeruginosa*. However, *Burkholderia cepacia*, *Stenotrophomonas maltophilia*, and *Aeromonas* spp. are distributed as follows: 5 (2.6%), 8 (4.1%), and 2 (1%), respectively. While 95 strains (48.5%) were Gram positive consisting of *Staphylococcus aureus* with an incidence of 49.5% (47/95), 33.7% (32/95) of coagulase-negative *Staphylococcus* (CoNS), 15.8% of *Bacillus* strains (15/95), and a single strain of *Streptococcus* belonging to the Lancefield serological group D.

### 3.2. Antimicrobial Sensitivity Pattern of the Isolates

Table 2 illustrates the resistance profile of GNB with respect to several families of antibiotics. *Enterobacteriaceae* strains showed high resistance to ampicillin and amoxicillin-clavulanic acid, with resistance rates of 90.3% and 83.9%, respectively, while they showed total sensitivity to imipenem and 32.3% (20/62) produced an extended-spectrum beta-lactamase (ESBL). It is also concluded, according to the current definition of MDR, that 44.4% (8/18) of *Pseudomonas* and 50% (3/6) of *Acinetobacter* are MDR, that is, strains resistant to at least three classes of antibiotics [14], while 44.7% (21/47) of *S*. *aureus* strains were methicillin resistant.

## 4. Discussion

The role of the environment in hosting and transmitting MDR organisms has become clearer through a series of publications linking environmental contamination to the increased risk of hospital-acquired infections. The incidence of antimicrobial resistance is also increasing and resulting in higher morbidity and mortality associated with nosocomial infections [15].

In this study, the overall prevalence of bacterial contamination in the different services is 88.4%. At the national level, Saouide el Ayne et al. reported a contamination rate of 96.3%. It is interesting to note here that the same rate was found by El Ouali Lalami et al. at the Fez city regional hospital. Elsewhere, similar studies conducted in Nigeria and Ethiopia reported lower rates of positivity at 46.7% and 39.6%, respectively [16–19]. Beyond Africa, a research carried out in Taiwan reflected a prevalence of 63.5%. Another study conducted in seven hospitals in Iran revealed 57%, and a research in a surgical setting at the Western General Hospital, in Scotland, in 2018, indicated a rate of 95.7% [20–22]. This contamination thus varies, qualitatively and quantitatively over time, from one institution to another and within the same establishment, and according to the services, patients, care, and techniques practiced. This difference is also due to the high sensitivity of the method for isolating bacterial colonies subsequent to an enrichment step [10].

Along with the quality of the organic cleaning applied, surface contamination depends on many factors related to microorganisms and their viability (survival) in the environment favored by the formation of biofilms. The latter varies according to the type of bacteria and the nature of the contaminated surfaces. This environmental impact was favored by the formation of biofilms on the surfaces [23]. The bacteria found on the surfaces also depend on the quality of the air because the particles suspended in the air will inevitably end up deposited on the surfaces and all the more quickly when they are larger. Thus, besides the quality of the biocleaning, the surface samples of a room will reflect the efficiency or the failures of an air treatment system.

The acquisition of resistance to amoxicillin/clavulanate, the antibiotic with a very strong prescription in Morocco, is a worldwide phenomenon reported at substantially variable rates. In this study, resistance to amoxicillin-clavulanate (Augmentin) was 83.9%. A similarly high rate (76.4%) was found in Cameroon [24].

Concerning the strains of *Enterobacteriaceae* involved in the contamination of the different surfaces, 32.3% (20/62) produced ESBL. This rate is close to that reported by the study conducted at the Cheikh Zaid Hospital, in Rabat, Morocco, in 2012, which found that 25.93% of ESBLs were isolated from medical equipment samples [25]. The total sensitivity of strains to imipenem may be due to the fact that this drug is not an antibiotic commonly used in human medicine.

For *S*. *aureus* strains, 44.7% were identified as MRSA. This prevalence remains lower compared to that recorded by Worku et al., which is 73.7% [26]. This bacterium is one of the most commonly implicated agents in nosocomial infections. Recent estimates suggest MRSA causes between 11,000 and 18,000 deaths and 80,000 invasive infections in the US annually [27, 28]. The proteins present in their cell walls for biofilm evasion of host immune responses and tissue-adhering abilities have enabled these organisms to survive and flourish in nature (https://bmcinfectdis.biomedcentral.com/articles/10.1186/s12879-018-2980-5) [29]. The significant presence of CoNS at the surface level (16.3%) could increase the danger of their involvement in nosocomial diseases, which is increasingly reported in the literature and which is accentuated by their MDR, which is also proven [30].

There is also a relatively high prevalence of resistance (50%) in strains of *Acinetobacter* spp. Exposure of strains of this species to the selective pressure of potent antibiotics has gradually led to the global prevalence of strains resistant to all *β*-lactamins and carbapenems. This resistance is due to combined mechanisms, generally including impermeability of the cell membrane, increased expression of efflux pumps, and the production of enzyme *β*-lactamase of several types [31].

In the medical services, 95% (38/40) of the sites were contaminated with 8 E-ESBL, 9 MRSA, and 5 MDR *P*. *aeruginosa*, which, on the one hand, could be related to the high activity taking place in these services, and on the other, to the high prescription of antibiotics, especially for patients hospitalized following diabetic foot infections. In fact, when patients are colonized, and especially when there is a patient infection, their immediate environment is generally highly contaminated by MDR bacteria as well as by those with frequent natural antibiotic resistance [32, 33].

For the operating theatre, the level of contamination discovered is also high (75%) and thus constitutes a high risk of occurrence of a surgical site infection (ISO), even worse if 38.1% (8/21) of MRSA were isolated from the same service. Although the role played by surface contamination in the genesis of ISOs is difficult to assess, a survey of the prevalence of nosocomial infections conducted at the Hassan II University Hospital, in Fez, Morocco, revealed that ISOs were mostly affected by nosocomial infections (46%) [34].

The evolution of the Gram-negative bacilli (GNB) resistance antibiotics reveals that MDR has remained, however, much more worrying in recent years. In February 2017, the WHO classified *P*. *aeruginosa*, *Acinetobacter baumannii*, and E-ESBL as the most critical pathogens from the point of view of antimicrobial resistance and for which the research and development of new antibiotics is a priority [35]. This group is composed of bacteria resistant to many extended-spectrum antibiotics including carbapenems, widely used to fight against MDR bacteria in hospitals.

The fact is that the emergence of MDR at the hospital is a real danger to public health if nothing is done to limit this spread. It may lead to epidemics difficult to eradicate, particularly with the bidirectional translocation of these bacteria between the hospital and the community. Furthermore, by mastering the method of predisinfection and the cleaning of reusable medical devices, the population of microorganisms can be reduced and the subsequent stage of disinfection or sterilization of the equipment can be facilitated. Also, extremely close attention should be paid to hospital hygiene training: hand hygiene, the use of personal protective equipment, and regulatory requirements, such as the safe disposal of medical waste and the safe handling of laundry [36].

It is noteworthy here that owing to rather limited variables in this study, it was not possible to determine the factors associated with the significant contamination of the hospital environment. Similarly, antimicrobial susceptibility patterns have also been determined with respect to just bacteria considered to present a high risk for patients to develop nosocomial diseases. Thus, we recommend extending the determination of the susceptibility profile to all bacteria associated with surface contamination and the development of their characteristics in order that we may be able to decipher the genes involved in antimicrobial resistance and their transmission mode.

## 5. Conclusion

In brief, in view of the results of this first study carried out at the hospital in Mohammedia and owing to the high levels of resistance in all types of isolated bacteria, which affect different services, it is important and urgent to assess and strengthen infection-prevention practices. Moreover, current hand-hygiene guidelines and recommendations for surface cleaning and disinfection should be thoroughly followed and adhered to in managing outbreaks due to these emerging pathogens.

## Figures and Tables

**Table 1:** Total number of screened equipment and surfaces and organisms detected.

Gram	Sampling site								
	Organism detected	Floor and wall (50)	Patient beds (46)	Bedside table (15)	Door/window handle (35)	Sinks and taps (10)	Hospital equipment (24)	Other items (20)	Total = 200
Gram + Total = 95 (48.5%)	*S*. coagulase negative	12	7	—	4	2	3	4	32 (16.3%)
	*Bacillus* spp.	8	3	—	2	—	—	2	15 (7.7%)
	*S*. *aureus*	12	11	4	10	—	6	4	47 (24%)
	*Streptococcus* spp.	—	01	—	—	—	—	—	01 (0.5%)
	*Enterobacter* spp.	2	7	1	4	1	3	1	19 (18.8%)
	*Klebsiella* spp.	3	1	2	4		4	4	18 (9.2%)
	*Pseudomonas aeruginosa*	3	6	—	2	2	2	3	18 (8.1%)
	*Serratia* spp.	2	2	2	4	—	—	—	10 (5.1%
	*Proteus* spp.	4	—	2	0	—	1	1	8 (4.1%)
	*Stenotrophomonas maltophilia*	2	—	0	3	—	—	2	7 (3.6%)
	*Acinetobacter* spp.	3	—	—	—	1	—	2	6 (3.1%)
	*Burkholderia cepacia*	4	—	—	2	—	—	—	6 (3.1%)
	*Citrobacter* spp.	1	—	—	—	1	3	—	5 (2.6%)
	*E*. *coli*	—	1	1	—	—	—	—	2 (1%)
	*Aeromonas* spp.	—	—	—	—	2	—	—	2 (1%)
	Total = 196	**56** ^*∗*^	**39** ^*∗*^	**12**	**35** ^*∗*^	**9**	**22** ^*∗*^	**23** ^*∗*^	**196**

Other items: objects that are not used directly to the patient such as scialytics, trolleys, and some closet; —, no detection of specified organism in this particular object; ^*∗*^mixed growth of bacteria.

**Table 2:** Antibiotic susceptibility pattern of GNB and *Staphylococcus aureus*.

Antibiotics	*Enterobacteriaceae* (*n* = 62)								
	*Enterobacter* spp. (*n* = 19)	*Klebsiella* spp. (*n* = 18)	*Serratia* spp. (*n* = 10)	*Proteus* spp. (*n* = 8)	*Citrobacter* spp. (*n* = 5)	*E. coli* (*n* = 2)	*P. aeruginosa* (*n* = 18)	*Acinetobacter* (*n* = 6)	*S. aureus* (*n* = 47)
Penicillin	—	—	—	—	—	—	—	—	25 (53.2%)
Ampicillin	19 (100%)	18 (100%)	10 (10%)	5 (63%)	4 (80%)	1 (50%)	—	—	—
Amoxicillin-clavulanic acid	18 (95%)	15 (83.3%)	10 (100%)	3 (38%)	5 (100%)	1 (50%)	—	—	—
Piperacillin	7 (36.8%)	10 (55.6%)	10 (100%)	00	3 (60%)	1 (50%)	8 (44.4%)	5 (83%)	—
Piperacillin + tazobactam	4 (21%)	1 (5.6%)	2 (20%)	00	1 (20%)	00	4 (22.2%)	3 (50%)	—
Ticarcillin	11 (57.9%)	13 (72.2%)	9 (90%)	00	3 (60%)	00	8 (44.4%)	5 (83%)	—
Ceftriaxone	11 (57.9%)	5 (27.8%)	2 (20%)	4 (50%)	1 (20%)	00	—	4 (67%)	—
Ceftazidime	8 (42.1%)	6 (33.3%)	2 (20%)	0 (13%)	1 (20%)	1 (50%)	8 (44.4%)	4 (67%)	—
Cefepime	00	00	00	00	00	00	00	00	—
Cefoxitin	8 (42.1%)	6 (33.3%)	8 (80%)	00	1 (20%)	2 (100%)	—	—	21 (44.7%)
Erythromycin	—	—	—	—	—	—	—	—	26 (55.3%)
Imipenem	00 (00%)	00 (0%)	00 (0%)	00	00	00	1 (5.6%)	3 (50%)	—
Chloramphenicol	6 (31.6%)	10 (55.6%)	00 (0%)	5 (63%)	2 (40%)	00	—	—	21 (44.7%)
Gentamicin	10 (52.6%)	1 (5.6%)	8 (80%)	2 (25%)	1 (20%)	00	8 (44.4%)	2 (33.3%)	19 (40.4%)
Kanamycin	—	—	—	—	—	—	—	—	27 (57.4%)
Tetracycline	14 (73.7%)	7 (38.9%)	7 (70%)	2 (25%)	1 (20%)	1 (50%)	—	5 (83%)	25 (53.2%)
Ciprofloxacin	5 (26.3%)	6 (33.3%)	5 (50%)	7 (88%)	3 (60%)	00	10 (55.6%)	2 (33.3%)	29 (61.7%)
Levofloxacin	4 (21%)	4 (22.2%)	2 (20%)	1 (13%)	1 (20%)	00	8 (44.4%)	5 (83%)	6 (12.8%)
Fusidic acid	—	—	—	—	—	—	—	—	16 (34.04%)
Trimethoprim-sulfamethoxazol	9 (47.37%)	8 (44.4%)	2 (20%)	3 (38%)	2 (40%)	2 (100%)	—	—	19 (40.4%)
ESBL	8 (42.11%)	9 (50%)	2 (20%)	00	00	1 (50%)	—	—	—

—: not applicable; ESBL: extended-spectrum beta-lactamase.

## Data Availability

All relevant data generated and analyzed during this study are included in this manuscript.
